# The Itch Cured in Two Hours

**Published:** 1852-05

**Authors:** 


From the London Lancet.

*The Itch cured in two hours.*

Dr. Bazin, Physician of the Hospital Saint Louis, of Paris, introduced not long ago, a notable improvement in the treatment of the itch, since he succeeded in curing the disease in *two days* by general frictions with the sulphur ointment. Dr. Hardy, who succeeded Dr. Bazin in the Scabies wards of the same hospital, has, however, considerably curtailed this already short time; he cures his patients in *two hours*. The method is described as follows:

Patients are no longer admitted *into* the house for the treatment of the itch, as two hours suffice to render contagion impossible and the recovery almost certain. The patient is put into a warm bath, and rubbed for an hour with yellow soap; he then passes into a clean bath, where he continues to cleanse his skin for another hour. After leaving this bath he is taken to a particular room fitted for the purpose, and, with the aid of one of his fellow-sufferers, he is rubbed all over for half an hour with the following ointment: Axunge eight parts, flour of sulphur two parts, carbonate of potash one part. After this friction, the patient is examined and sent away cured, though sometimes pretty numerous vesicles on the hand and elsewhere, remain unaltered. Dr. Hardy states that out of one hundred cases he has hardly had two or three relapses. The number of itch patients had considerably diminished, as none are now turned away for want of room; and the disease has thus spread with much less rapidity.

